# Supplementary material for: The molecular cytoarchitecture of the adult mouse brain
Source: Nature. 2023 Dec 13;624(7991):333–42. doi: 10.1038/s41586-023-06818-7 (PMC10719111; doi:10.1038/s41586-023-06818-7)
Supplement: Supplementary file 1 — Supplementary Methods [file 41586_2023_6818_MOESM1_ESM.pdf]

nature portfolio

https://doi.org/10.1038/s41586-023-06818-7

Supplementary information

The molecular cytoarchitecture of the adult mouse brain

In the format provided by the
authors and unedited

The molecular cytoarchitecture of the adult mouse brain

Jonah Langlieb*[1], Nina S. Sachdev*[1], Karol S. Balderrama[1], Naeem M. Nadaf[1], Mukund Raj[1], Evan Murray[1], James T. Webber[1], Charles Vanderburg[1], Vahid Gazestani[1], Daniel Tward[2], Chris Mezias[3], Xu Li[3], Katelyn Flowers[1], Dylan M. Cable[1,4], Tabitha Norton[1], Partha Mitra[3], Fei Chen*[1,5], Evan Z. Macosko*[1,6]

1. Broad Institute of Harvard and MIT, Cambridge, MA USA
2. Departments of Computational Medicine and Neurology, University of California Los Angeles, Los Angeles, CA USA
3. Cold Spring Harbor Laboratory, Cold Spring Harbor, NY USA
4. Department of Electrical Engineering and Computer Science, Massachusetts Institute of Technology, Cambridge, MA USA
5. Harvard Stem Cell and Regenerative Biology, Cambridge, MA USA
6. Department of Psychiatry, Massachusetts General Hospital, Boston, MA USA

1 Supplementary Methods

1.1 Discovery of combinatorial marker genes needed to distinguish snRNA-seq cell types

To find the minimally-sized gene lists which allowed us to distinguish one cell type from the others in the dataset, we framed the question as a set covering problem. In the set cover problem, we find the smallest subfamily of a family of sets that can still cover all the elements in a universe set. For our use case, we examine a given cell type A and define a family of sets, one set per gene. The set S_g, corresponding to the gene g, contains all the other cell types which are 'distinguishable' (defined below) from A using the gene g. In this way, the minimal set cover of this family of sets gives the smallest subset of genes which distinguishes A from all the other cell types in the dataset (i.e. the universe set).

To determine if a gene g is 'distinguishable' between two cell types Aand B, first we define $Z_{A,g}$ and $Z_{B,g}$to be the percent of cells in cluster A and cluster B with nonzero values of gene g, respectively. Then we say A is 'distinguishable' from B using g, denoted $D(g,A,B) = 1$, when $Z_{A,g} \geq 0.25$ and $\Big((Z_{A,g} - Z_{B,g}) \geq 0.5$ or $Z_{B,g} \leq 0.05\Big)$. Otherwise, we say A is "indistinguishable" from B using gene g, denoted as $D(g,A,B) = 0$.

Then to solve each cell type's minimum set cover to optimality (or to prove it is infeasible), we phrase this problem as a mixed integer linear programming (MILP) optimization problem.

We'll consider one cell type A of which we want to find the set cover. Given the C other cell types: $c_1, c_2, ..., c_C$; which express G genes: $g_1, g_2, ..., g_G$; we create a matrix M of dimension $(C \times G)$ where $M_{i,j} = D(g_j, A, C_i)$.

We formulate our set cover model as follows:

Decision variable. The main variable we want to optimize is the choice of which genes to select for the set cover. So, we define the binary decision variable:

$$U_j = \begin{cases} 1, \text{If gene } g_j \text{ is selected} \\ 0, \text{ otherwise.} \end{cases} ; j = 1, 2, \ldots, G.$$

Constraints. We then define $X = MU$, a C-length vector where X_i gives how many times cell type c_i was covered by the genes selected in U. For U to give a proper set cover (i.e. every cell type is covered at least once by the selected genes), we constrain the model such that

$$X_i \geq 1;\ i = 1,\ 2, \ldots,\ C.$$

Note that if we want to exclude cell types some cell types C_a and C_b from the optimization (e.g. they are the two nearest neighbors to A) we can subset X to exclude these two rows. Equivalently, we instead generate a one-hot encoding for the cell types to be excluded with

$$E_i = \begin{cases} 1, \text{If cell type } c_i \text{ is excluded} \\ 0, \text{ otherwise.} \end{cases} ; i = 1, 2, \ldots, C$$

and redefine $X = (MU)\ +\ E$.

Objective. Our goal is to minimize the number of genes chosen. Formally

$$\min \sum_{j=1}^{G} U_j.$$

We can define this mixed integer linear model programmatically using the JuMP domain-specific modeling language in Julia [1, 2]. We optimized using the HiGHS open source solver [3] or the IBM ILOG CPLEX commercial solver v22.1.0.0 [4]. We repeated this optimization for every cell type in the dataset. We also group the cell types by main region (detailed above) and repeat this optimization considering only cell types within each regional group.

To enumerate all possible gene lists, for use in obtaining the minimum-sized gene list (below), we used the IBM CPLEX solver with a 5 hour time-limit over two threads and the following parameters:

```
mip pool absgap 0
mip pool intensity 4
mip limits populate 10000000
```

1.2 Creation of minimum-sized collated gene list

By default, the CPLEX optimizers stop after finding one optimal solution (i.e. minimally sized gene list). But, upon loosening this restriction, we found that most cell types had multiple distinct equally-optimal solutions. As an example, for the cell type Ex_Pitx2_Zbtb7c_3, its minimally sized gene lists are of length 3 but with many different variations, such as {*Foxd3*, *Pitx2*, *Tmem258*} and {*Foxd3*, *Pitx2*, *Tmem126b*}, which both satisfy the distinguishability criteria and only vary by the exact transmembrane protein chosen.

We wanted to investigate the extent to which genes repeatedly appear across these solution-sets and to examine whether these repeatedly occurring genes are enriched within any Gene Ontology (GO) classification [5]. So we again used a set cover approach, and leveraged this solution redundancy to identify the most succinct set encompassing at least one gene list from each cell type. With this method we found a ∼25% reduction in the size of the encompassing gene list, as opposed to taking the union of first-discovered gene lists returned by the default solvers.

To express this collated gene list as a set cover problem, we first define the G expressed genes: $g_1, g_2, \ldots,\ g_G$. Using the above exhaustive enumeration using IBM CPLEX, we obtain a list of equally-optimal (equally-small) gene sets for each of the C cell types: $c_1, c_2, \ldots,\ c_C$. We define $N_1, N_2, \ldots, N_C$ to be how many gene lists each cluster has. In total we have $L = \sum_{i=1}^{C} N_i$ gene lists, enumerated as $l_1,\ l_2, \ldots,\ l_L$. Note that lists $(l_1, l_2, \ldots,\ l_{N_1})$ all come from cell type c_1, lists $(l_{N_1+1},\ l_{N_1+2}, \ldots,\ l_{N_1+N_2})$ all come from cell type c_2 and so on. For ease of further reference, we will use $i|k$ to denote the index of the k'th gene list for cell type C_i in the whole L-length gene list. For example, $1|1 = 1$ (the first list of the first cell type is in position 1), $C|N_C = L$ (the last list of the last cell type is in the final or L'th position), and $2|1 = N_1 + 1$.

We can encode the individual gene membership for each gene list with a $(L \times G)$ matrix M where $M_{i,j}$ gives whether gene g_j is a member of list l_i.

We define our MILP model such that:

Decision Variables. The main binary decision variable we want to optimize is again

$$U_j = \begin{cases} 1, \text{If gene } g_j \text{ is selected} \\ 0, \text{otherwise.} \end{cases} ; j = 1, 2, \ldots, G$$

In this case, we need an additional decision variable for each of the L gene lists encoding whether it was completely covered by the genes selected in U. As we will see, this allows us to require at least one gene list to be completely covered. We define the binary variable

$$T_k = \begin{cases} 1, \text{If all the genes in } \ell_k \text{ are covered} \\ 0, \text{otherwise.} \end{cases} ; k = 1, 2, \ldots, L.$$

Constraints. We define $X = MU$, which is a L-length vector where X_i gives how many genes in l_i are covered by the genes selected in U.

For each cell type C_i we'll create different constraints. Note first that C_i has N_i different gene lists $l_{i|1}, l_{i|2}, \ldots,\ l_{i|N_i}$ all of the same (optimal) length. We'll define that length as S_i.

We require at least one of these gene list to be fully covered so constrain

$$\sum_{k=1}^{N_i} T_{i|k} \geq 1.$$

Then we want to enforce the requirements of decision variable T. So, for a gene list $l_{i|k}$, if all the genes in this set are covered by the chosen genes from U, then $T_{i|k} = 1$. Using the counterfactual, we see that if $T_{i|k} = 0$ then some number of genes in $l_{i|k}$ are *not* covered by the genes from U. In fact, we have an upper bound on this number, because all of the gene lists being examined are of size S_i, so at most S_i genes are missing from being covered by U in $T_{i|k}$.

Given a list $l_{i|x}$ where $T_{i|x} = 1$, for it actually to be fully covered by the non-zero elements of U, we require $X_{i|x} \geq S_i$. For a list $l_{i|y}$ where $T_{i|y} = 0$, since it is already not fully covered by the non-zero elements of U, we can ignore its corresponding $X_{i|y}$. One way of ignoring this set, while keeping the broader constraint in place for optimization, is by adding the upper bound of missing genes, S_i, since $X_{i|y} + S_i \ \geq S_i$ is true by inspection ($X_{i|y} \ \geq 0$ by definition). Putting this together we get the constraint

$$X_{i|k} + (S_i * (1 - T_{i|k})) \geq S_i;\ \ k = 1, 2, \ldots, N_i.$$

We repeat this constraint for every cluster C_i and for each cluster grouped by the main region.

References

[1] Bezanson, J., Edelman, A., Karpinski, S., Shah, V.B.: Julia: A Fresh Approach to Numerical Computing (2017)

[2] Dunning, I., Huchette, J., Lubin, M.: JuMP: A Modeling Language for Mathematical Optimization (2017)

[3] Huangfu, Q., Hall, J.A.J.: Parallelizing the dual revised simplex method (2018)

[4] Cplex, I.I.: V22. 1: User's manual for CPLEX. International Business Machines Corporation

[5] Ashburner, M., Ball, C.A., Blake, J.A., Botstein, D., Butler, H., Cherry, J.M., Davis, A.P., Dolinski, K., Dwight, S.S., Eppig, J.T., Harris, M.A., Hill, D.P., Issel-Tarver, L., Kasarskis, A., Lewis, S., Matese, J.C., Richardson, J.E., Ringwald, M., Rubin, G.M., Sherlock, G.: Gene ontology: tool for the unification of biology. the gene ontology consortium. Nat. Genet. **25**(1), 25–29 (2000)
