# Supplementary material for: The molecular cytoarchitecture of the adult mouse brain
Source: Nature. 2023 Dec 13;624(7991):333–42. doi: 10.1038/s41586-023-06818-7 (PMC10719111; doi:10.1038/s41586-023-06818-7)
Supplement: Supplementary file 2 — Reporting Summary [file 41586_2023_6818_MOESM2_ESM.pdf]

nature portfolio

Corresponding author(s): Evan Macosko, Fei Chen

Last updated by author(s): 10-16-2023

Reporting Summary

Nature Portfolio wishes to improve the reproducibility of the work that we publish. This form provides structure for consistency and transparency in reporting. For further information on Nature Portfolio policies, see our Editorial Policies and the Editorial Policy Checklist.

Statistics

For all statistical analyses, confirm that the following items are present in the figure legend, table legend, main text, or Methods section.

n/a	Confirmed	
☐	☒	The exact sample size (*n*) for each experimental group/condition, given as a discrete number and unit of measurement
☐	☒	A statement on whether measurements were taken from distinct samples or whether the same sample was measured repeatedly
☐	☒	The statistical test(s) used AND whether they are one- or two-sided<br>*Only common tests should be described solely by name; describe more complex techniques in the Methods section.*
☒	☐	A description of all covariates tested
☐	☒	A description of any assumptions or corrections, such as tests of normality and adjustment for multiple comparisons
☐	☒	A full description of the statistical parameters including central tendency (e.g. means) or other basic estimates (e.g. regression coefficient) AND variation (e.g. standard deviation) or associated estimates of uncertainty (e.g. confidence intervals)
☐	☒	For null hypothesis testing, the test statistic (e.g. *F*, *t*, *r*) with confidence intervals, effect sizes, degrees of freedom and *P* value noted<br>*Give P values as exact values whenever suitable.*
☒	☐	For Bayesian analysis, information on the choice of priors and Markov chain Monte Carlo settings
☒	☐	For hierarchical and complex designs, identification of the appropriate level for tests and full reporting of outcomes
☒	☐	Estimates of effect sizes (e.g. Cohen's *d*, Pearson's *r*), indicating how they were calculated

Our web collection on statistics for biologists contains articles on many of the points above.

Software and code

Policy information about availability of computer code

Data collection	Mouse raw sequencing data was processed, aligned and converted to digital gene expression matrix format by CellRanger v.3.0.2 (available from 10x Genomics) with default settings. We used CellBender v3-alpha to remove cells contaminated with ambient RNA. For Slide-seq data, the sequenced reads were aligned to GRCm39.103 reference and processed using the Slide-seq tools pipeline (https://github.com/MacoskoLab/slideseq-tools) to generate the gene count matrix and match the bead barcode between array and sequenced reads.
Data analysis	All analysis and code used for data clustering, integration, and analysis is available at https://github.com/MacoskoLab/brain-atlas.

For manuscripts utilizing custom algorithms or software that are central to the research but not yet described in published literature, software must be made available to editors and reviewers. We strongly encourage code deposition in a community repository (e.g. GitHub). See the Nature Portfolio guidelines for submitting code & software for further information.

Data

Policy information about availability of data

All manuscripts must include a data availability statement. This statement should provide the following information, where applicable:

- Accession codes, unique identifiers, or web links for publicly available datasets
- A description of any restrictions on data availability
- For clinical datasets or third party data, please ensure that the statement adheres to our policy

An interactive portal is available at www.braincelldata.org, where gene expression data and spatial localizations of individual cell types can be visualized in a number

of ways. Raw single-nucleus RNA-seq and Slide-seq data is available in the NeMO Archive (www.nemoarchive.org). The snRNA-seq clustering algorithm is available in the repository www.github.com/MacoskoLab/brain-atlas. This code was built on top of several existing packages: numpy, scipy, dask, pynndescent, leidenalg, igraph, zarr, and their dependencies.

Research involving human participants, their data, or biological material

Policy information about studies with human participants or human data. See also policy information about sex, gender (identity/presentation), and sexual orientation and race, ethnicity and racism.

Reporting on sex and gender	Not applicable
Reporting on race, ethnicity, or other socially relevant groupings	Not applicable
Population characteristics	Not applicable
Recruitment	Not applicable
Ethics oversight	Not applicable

Note that full information on the approval of the study protocol must also be provided in the manuscript.

Field-specific reporting

Please select the one below that is the best fit for your research. If you are not sure, read the appropriate sections before making your selection.

☒ Life sciences ☐ Behavioural & social sciences ☐ Ecological, evolutionary & environmental sciences

For a reference copy of the document with all sections, see nature.com/documents/nr-reporting-summary-flat.pdf

Life sciences study design

All studies must disclose on these points even when the disclosure is negative.

Sample size	No statistical methods were used to predetermine sample size for animals used to generate transcriptomic data; sample sizes were selected to ensure multiple replicates, in each region, for each sex.
Data exclusions	For transcriptomic data, pre-established exclusion criteria of a minimum of 500 UMI/nucleus and a maximum of 1% mitochondrial nUMIswere used, as these were standard thresholds used in previous studies.
Replication	Clusters generated from the transcriptomic data were examined to ensure that multiple technical replicates contributed to individual clusters. Analyses relevant to replication can be found in Extended Data Figure 1.
Randomization	No differential experimental treatments were applied to individuals or samples in this study.
Blinding	Blinding was not relevant in this study as no differential experimental treatments were applied.

Reporting for specific materials, systems and methods

We require information from authors about some types of materials, experimental systems and methods used in many studies. Here, indicate whether each material, system or method listed is relevant to your study. If you are not sure if a list item applies to your research, read the appropriate section before selecting a response.

Materials & experimental systems

n/a	Involved in the study	
☒	☐	Antibodies
☒	☐	Eukaryotic cell lines
☒	☐	Palaeontology and archaeology
☐	☒	Animals and other organisms
☒	☐	Clinical data
☒	☐	Dual use research of concern
☒	☐	Plants

Methods

n/a	Involved in the study	
☒	☐	ChIP-seq
☒	☐	Flow cytometry
☒	☐	MRI-based neuroimaging

Animals and other research organisms

Policy information about studies involving animals; ARRIVE guidelines recommended for reporting animal research, and Sex and Gender in Research

Laboratory animals	Mouse transcriptomic data was generated from adult female and adult male mice (60 days old; C57BL/6J, Jackson Labs). Specific data about the mice used can be found in Extended Data Figure 1.
Wild animals	No wild animals were used in the study.
Reporting on sex	Both male and female mice were jointly used in the generation of the single cell clustering data.
Field-collected samples	No field-collected samples were used in the study.
Ethics oversight	All procedures involving animals were conducted in accordance with the US National Institutes of Health Guide for the Care and Use of Laboratory Animals under protocol number 1115-111-18 and approved by the Massachusetts Institute of Technology Committee on Animal Care.

Note that full information on the approval of the study protocol must also be provided in the manuscript.

Plants

Seed stocks	N/A
Novel plant genotypes	N/A
Authentication	N/A
